# Supplementary material for: Shifting meiotic to mitotic spindle assembly in oocytes disrupts chromosome alignment
Source: EMBO Rep. 2018 Jan 12;19(2):368–81. doi: 10.15252/embr.201745225 (PMC5797964; doi:10.15252/embr.201745225)
Supplement: Supplementary file 1 — Expanded View Figures PDF [file EMBR-19-368-s001.pdf]

Expanded View Figures

Figure EV1. Endogenous HSET and slightly overexpressed exogenous HSET localize to the spindle throughout meiosis I.

A Immunofluorescence showing endogenous HSET (left panel), exogenous HSET WT (middle panel), and exogenous HSET N593K (right panel). Endogenous and exogenous HSET display the same localization: they all localize on the spindle, as observed here on fixed oocytes at NEBD+4h30 (HSET antibody: green; DNA: blue). Note that the spindle is elongated in HSET WT expressing oocytes. Scale bar 10 μm.

B Time-lapse spinning disk confocal microscopy of an oocyte expressing GFP-HSET WT (green) and Histone-RFP (blue). GFP-HSET WT localizes on the spindle throughout meiosis I. The white asterisk marks a chromosome outside of the metaphase plate. Scale bar 5 μm.

C HSET total fluorescence intensity measured in the whole cell for fixed oocytes at NEBD+4h30 expressing HSET WT or not (Ctrl). Data are represented as mean ± SD. The ratio of total HSET WT overexpression is 1.6.

D HSET normalized fluorescence intensity measured on the spindle of fixed oocytes at NEBD+4h30 expressing HSET WT or not (Ctrl). Data are represented as mean ± SD. The ratio of HSET OE on the spindle is 4.2.

E Time-lapse spinning disk confocal microscopy of an oocyte expressing GFP-HSET N593K (green). GFP-HSET N593K displays a localization similar to endogenous or GFP-HSET WT on the spindle throughout meiosis I. Scale bar 10 μm.

F GFP total fluorescence intensity measured in the whole cell for live oocytes at NEBD+7h expressing GFP-HSET WT or GFP-HSET N593K. Data are represented as mean ± SD. Statistical significance of differences is assessed with a *t*-test: ***P*-value = 0.004.

© 2018 The Authors

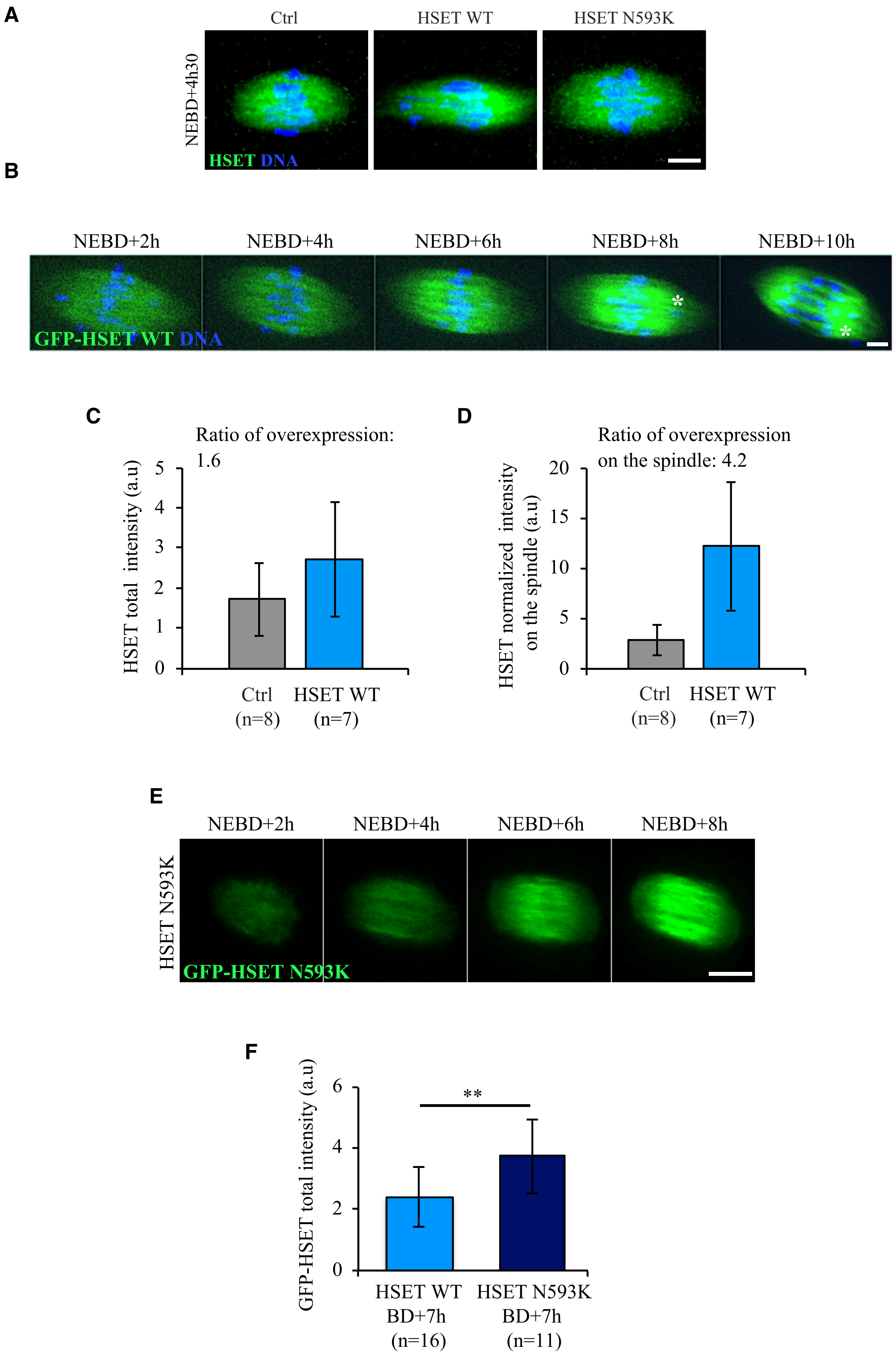

Figure EV1.

© 2018 The Authors

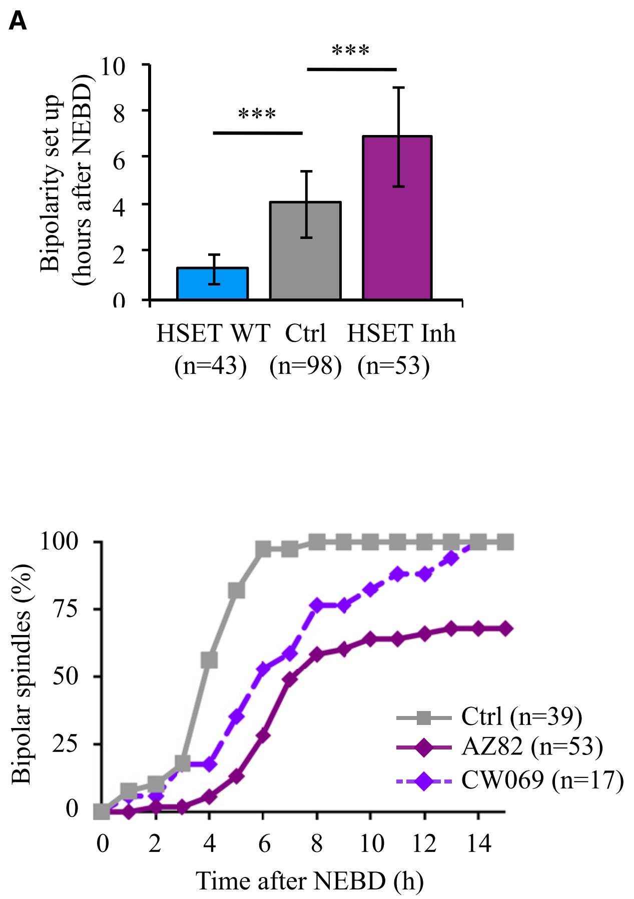

Figure EV2. HSET levels control the timing of spindle bipolarization.

A Histogram showing the mean time of bipolarization setup (data are represented as mean ± SD). Bipolarity was scored when two poles were distinguishable. The mean time of bipolarization setup for oocytes overexpressing HSET WT is 1 h and 19 min (blue bar), compared to 4 h and 3 min for controls (gray bar), ****P*-value < 0.0001, compared to 6 h and 55 min for oocytes inhibited for HSET (purple bar), ****P*-value < 0.0001. Statistical significance of differences is assessed with a Mann–Whitney test.

B Histogram showing the mean time of bipolarization setup in controls vs. HSET N593K expressing oocytes (data are represented as mean ± SD). Bipolarity was scored when two poles were distinguishable. The mean time of bipolarization setup for oocytes overexpressing HSET N593K is 2 h and 36 min (dark blue bar), compared to 3 h and 10 min for the controls (gray bar). Statistical significance of differences is assessed with a *t*-test: **P*-value = 0.034.

C Graph representing the kinetics of spindle bipolarization in controls (gray) vs. oocytes inhibited for HSET with AZ82 (purple) or CW069 (violet). The number of oocytes analyzed is written in parentheses.

D Histogram showing the mean time of bipolarization setup. Bipolarity was scored when two poles were distinguishable. Data are represented as mean ± SD. The mean time of bipolarization setup for controls (gray bar) is 4 h and 18 min, compared to 6 h and 55 min for oocytes inhibited for HSET with AZ82 (purple bar), compared to 7 h and 0 min for oocytes inhibited for HSET with CW069 (violet bar). Statistical significance of differences is assessed with a Mann–Whitney test: ***P*-value = 0.007, ****P*-value < 0.0001, not significant (n.s.) *P*-value = 0.929. The number of oocytes analyzed is written in parentheses.

Figure EV3. Spindle morphogenesis after perturbation of HSET levels.

A Principle of the automated 3D analysis of aMTOCs within the spindle. We developed a Fiji plug-in that converts images from spinning disk confocal live microcopy (here a spindle region magnification of an oocyte expressing GFP-EB3 (green) and mCherry-Plk4 (red) at NEBD+6h30, left panel) to binary images (middle panel), and then to 3D images (right panel).

B aMTOCs sorting in controls (gray dots) and oocytes overexpressing HSET WT (blue dots) at NEBD+1h30, +4h30, and +6h30. Each dot is one aMTOC. The vertical axis plots the aMTOCs volume, and the horizontal axis represents an hemi-spindle starting from the central spindle to the pole (as written on the scheme). The distance of aMTOCs to the closest spindle pole is normalized by the spindle length.

C Binary images corresponding to Fig 2A. Scale bar 10 µm.

D Quantification in 3D of the spindle length in controls (gray dots) and oocytes inhibited for HSET (purple dots) at NEBD+1h30, +4h30, and +6h30. Each dot represents an oocyte, the number of oocytes analyzed is written in parentheses. Statistical significance of differences is assessed with a Mann–Whitney test: **P*-value = 0.017, ***P*-value = 0.003, ****P*-value < 0.0001.

© 2018 The Authors

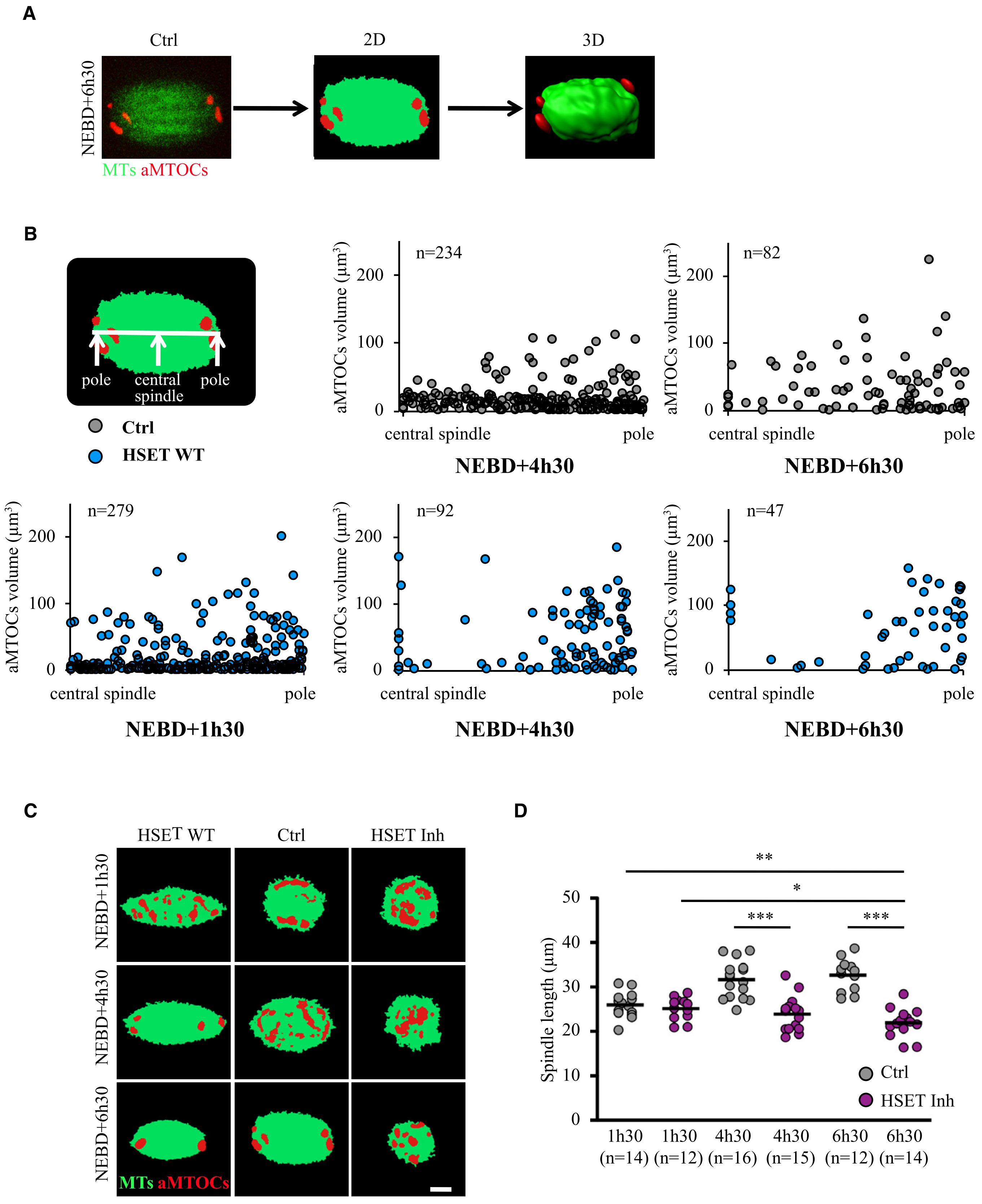

Figure EV3.

 © 2018 The Authors

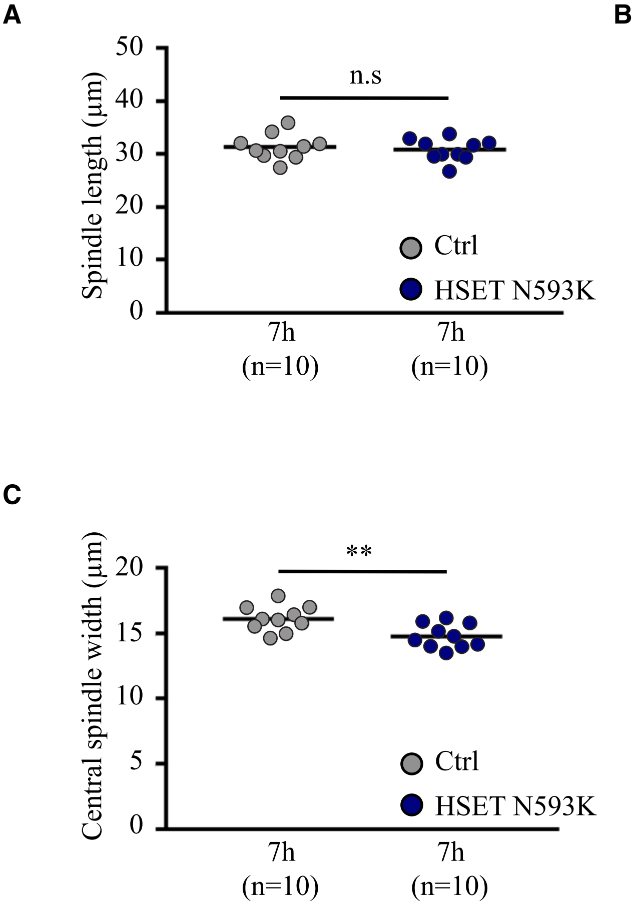

Figure EV4. HSET N593K overexpression does not alter spindle shape.

A–C Quantification in 3D of the spindle length (A) not significant (n.s.) *P*-value = 0.616, spindle pole width (B) not significant (n.s.) *P*-value = 0.175 and central spindle width (C) ***P*-value = 0.006 in oocytes overexpressing HSET N593K (dark blue dots) and controls (gray dots) at NEBD+7h. Each dot represents an oocyte, and the number of oocytes analyzed is written in parentheses. Statistical significance of differences is assessed with a Mann–Whitney test.

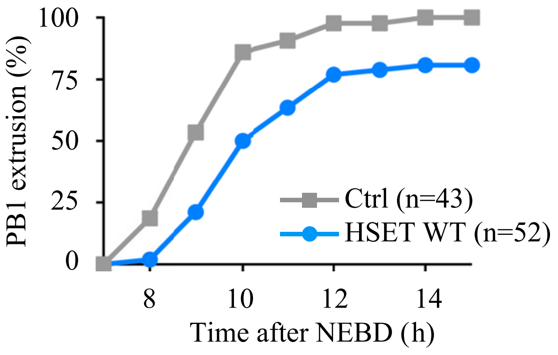

Figure EV5. Anaphase occurs with a modest delay in oocytes overexpressing HSET WT.

Graph representing the kinetics of polar body (PB1) extrusion in controls (gray) vs. HSET WT overexpressing oocytes (blue) obtained from three independent experiments.
